# The Continuum of Physiological Impairment during Treadmill Walking in Patients with Mild-to-Moderate COPD: Patient Characterization Phase of a Randomized Clinical Trial

**DOI:** 10.1371/journal.pone.0096574

**Published:** 2014-05-01

**Authors:** Denis E. O’Donnell, François Maltais, Janos Porszasz, Katherine A. Webb, Frank C. Albers, Qiqi Deng, Ahmar Iqbal, Heather A. Paden, Richard Casaburi, on behalf of the 205.440 investigators

**Affiliations:** 1 Queen’s University & Kingston General Hospital, Kingston, Ontario, Canada; 2 Centre de Recherche, Institut Universitaire de Cardiologie et de Pneumologie de Québec, Université Laval, Québec, Canada; 3 Rehabilitation Clinical Trials Center, Los Angeles Biomedical Research Institute at Harbor-UCLA Medical Center, Torrance, California, United States of America; 4 Boehringer Ingelheim Pharmaceuticals, Inc., Ridgefield, Connecticut, United States of America; 5 Pfizer Inc, New York, New York, United States of America; University Hospital Freiburg, Germany

## Abstract

**Background:**

To have a better understanding of the mechanisms of exercise limitation in mild-to-moderate chronic obstructive pulmonary disease (COPD), we compared detailed respiratory physiology in patients with COPD and healthy age- and sex-matched controls.

**Methods:**

Data were collected during the pre-treatment, patient characterization phase of a multicenter, randomized, double-blind, crossover study. Patients with COPD met Global Initiative for Chronic Obstructive Lung Disease (GOLD) 1 or 2 spirometric criteria, were symptomatic, and had evidence of gas trapping during exercise. All participants completed pulmonary function and symptom-limited incremental treadmill exercise tests.

**Results:**

Chronic activity-related dyspnea measured by Baseline Dyspnea Index was similarly increased in patients with GOLD 1 (n = 41) and 2 (n = 63) COPD compared with controls (n = 104). Plethysmographic lung volumes were increased and lung diffusing capacity was decreased in both GOLD groups. Peak oxygen uptake and work rate were reduced in both GOLD groups compared with controls (p<0.001). Submaximal ventilation, dyspnea, and leg discomfort ratings were higher for a given work rate in both GOLD groups compared with controls. Resting inspiratory capacity, peak ventilation, and tidal volume were reduced in patients with GOLD 2 COPD compared with patients with GOLD 1 COPD and controls (p<0.001).

**Conclusions:**

Lower exercise tolerance in patients with GOLD 1 and 2 COPD compared with controls was explained by greater mechanical abnormalities, greater ventilatory requirements, and increased subjective discomfort. Lower resting inspiratory capacity in patients with GOLD 2 COPD was associated with greater mechanical constraints and lower peak ventilation compared with patients with GOLD 1 COPD and controls.

**Trial Registration:**

ClinicalTrials.gov: **** NCT01072396

## Introduction

The majority of patients with chronic obstructive pulmonary disease (COPD) worldwide have mild-to-moderate disease severity [1], yet these patients are less extensively studied than those with severe disease. Population-based studies have shown that smokers with milder COPD have increased mortality [2], increased hospitalizations, decreased health-related quality of life [3], [4], increased activity-related dyspnea, and reduced daily physical activity levels compared with non-smoking healthy individuals [5]–[9]. Although evidence has recently emerged to support the link between dyspnea and activity restriction in mild COPD [10], [11], a better understanding of the underlying pathophysiology is needed to rationalize management strategies for symptomatic patients.

It is well established that apparently minor airflow obstruction (as measured by spirometry) may obscure widespread damage to the peripheral airways (<2 mm diameter), lung parenchyma, and pulmonary vasculature [12]. Previous studies have revealed vast pathophysiological heterogeneity in mild COPD and successfully quantified the extent of small airway dysfunction and pulmonary gas exchange abnormality at rest [13]–[15]. More recently, small studies have shown that these physiological perturbations are amplified during the stress of cycle ergometer exercise in mild COPD. Thus, compared with age-matched healthy controls, ventilatory requirements were consistently increased for a given metabolic load and gas trapping was increased, suggesting significant ventilation–perfusion abnormalities and small airway dysfunction, respectively [10], [11], [16], [17]. The current multicenter study extends previous investigations in mild-to-moderate COPD by providing comparisons of detailed pulmonary physiology in a large number of patients with Global Initiative for Chronic Obstructive Lung Disease (GOLD) spirometry grade 1 and 2 COPD [18] and healthy controls. We selected symptomatic patients (reflective of group B in the 2014 GOLD recommendations [18]) and evidence of dynamic lung hyperinflation during exercise, where pharmacologic treatment is more likely to be beneficial. The latter criterion (dynamic hyperinflation) was included because it is a marker of expiratory flow limitation during exercise in symptomatic patients with COPD who have a largely preserved forced expiratory volume in 1 s (FEV_1_) [10], [11], [16], [17]. Moreover, recent studies in symptomatic patients with mild-to-moderate COPD have shown that measureable dynamic hyperinflation was present in the vast majority of cases [10], [11], [14], [19]. In the current study, treadmill exercise was selected rather than cycling because it is potentially more reflective of common daily activity i.e., walking. Weight-bearing treadmill exercise is fundamentally different from weight-supported cycling in terms of the extent of metabolic loading, acid-base and pulmonary gas exchange abnormalities, and locomotor muscle recruitment pattern [20]–[22]. Therefore, it remains to be determined if mechanical and gas exchange derangements exist during walking in milder COPD, and if these are similar in magnitude and direction to those previously documented during cycle ergometry in this population.

Our main objective was to evaluate the continuum of physiological heterogeneity during rest and exercise in symptomatic patients with GOLD 1 and 2 COPD who had demonstrable gas trapping (inspiratory capacity [IC] decline ≥100 mL) during exercise. Therefore, we compared resting pulmonary function and ventilatory responses to treadmill exercise, including breathing pattern, operating lung volumes, and dyspnea and leg discomfort, in patients with GOLD 1 and 2 COPD and healthy controls matched for age and sex. This prospective physiological study represents the initial “patient characterization” phase of a randomized, placebo-controlled study designed to evaluate the efficacy of tiotropium in patients with mild-to-moderate COPD. Results of the treatment phase will be presented separately.

## Methods

The protocol for this trial and supporting CONSORT checklist are available as supporting information; see Protocol S1 and Checklist S1.

### Ethics Statement

The study was carried out in compliance with the approved protocol, the principles laid down in the Declaration of Helsinki version as of October 1996, and in accordance with the International Conference on Harmonisation Tripartite Guidelines for Good Clinical Practice. Written informed consent was obtained from all participants; the study protocol, informed consent, and patient information were reviewed and approved by the following local Institutional Review Boards/Independent Ethics Committees: Chesapeake Research Review, Inc. (Columbia, MD); The John F. Wolf, M.D. Human Subjects Committee of the Los Angeles Biomedical Research Institute at Harbor UCLA Medical Center (Torrance, CA); Partners Human Research Committee (Boston, MA); Western Institutional Review Board (Olympia, WA); Springfield Committee for Research Involving Human Subjects (Springfield, IL); Trustees of Dartmouth College, Dartmouth–Hitchcock Medical Center, Committee for the Protection of Human Subjects (Hanover, NH); Saint Francis Hospital and Medical Center Institutional Review Board (Hartford, CT); McGill University Health Center Research Ethics Office (Montreal, QC); Comité d’Éthique de la Recherche de I’Institut Universitaire de Cardiologie et de Pneumologie de Quebéc (Québec, QC); Queen’s University, Health Sciences and Affiliated Teaching Hospitals Research Ethics Board (Kingston, ON); and Centre Hospitalier de l’Université de Montreal (CHUM) Research Ethics Committee (Montreal, QC).

### Study Design

This was a multicenter, multinational (11 US and four Canada sites), randomized, double-blind, two-period crossover study (ClinicalTrials.gov identifier: NCT01072396). The current analysis covered the 2-week pre-treatment patient characterization phase of the study (Visits 1–3) in which patients with COPD were compared with control subjects. Participants completed symptom and activity assessments, pulmonary function tests, and an incremental cardiopulmonary exercise test (Visit 1) and constant work rate tests (Visits 2 and 3). Constant work rate test data are not presented here.

### Participants

Study participants were males and females, age ≥40 years, with a body mass index (BMI) 18–35 kg/m^2^. Patients with COPD met the following inclusion criteria: current or ex-smokers with >10 pack-year history; post-bronchodilator FEV_1_/forced vital capacity (FVC) <70% and FEV_1_≥50% of predicted normal at Visit 1; clinically stable as defined by no exacerbations in the previous 6 weeks and <3 exacerbations in the preceding year; symptomatic as defined by a Baseline Dyspnea Index (BDI) [23] focal score ≤9 and/or daily cough with sputum for 3 months per year during ≥2 consecutive years; and ≥100 mL decrease in IC during treadmill exercise, as evidence of dynamic lung hyperinflation. Decrease in IC was measured as the difference between the last acceptable IC maneuver during exercise and the average pre-exercise resting IC. To meet the inclusion criteria patients had to demonstrate ≥100 mL decrease in IC in two of three exercise tests during the pre-treatment patient characterization phase (incremental [Visit 1] or constant work rate [Visits 2 and 3]). A control group consisted of healthy subjects that were age- and sex-matched to the patients with COPD. Control subjects were non-smokers, i.e., no cigarettes in the previous 2 years and <1 pack-year smoking history. Subjects were excluded if they had contraindications to exercise, a condition that limited exercise ability, or if they required supplemental oxygen. Patients with COPD were categorized into two groups: GOLD 1 (post-bronchodilator FEV_1_/FVC <0.7 and FEV_1_ ≥80% of predicted normal) and GOLD 2 (post-bronchodilator FEV_1_/FVC <0.7 and 50%≤ FEV_1_ <80% of predicted normal).

Respiratory medications permitted during the baseline period included: albuterol meter-dose inhaler (100 µg/puff) as needed (withheld 8 h prior to each visit); short-acting anticholinergics (withheld 8 h prior to each visit); long-acting β_2_-agonists (withheld 36 h prior to each visit); antihistamines, mucolytics, and antileukotrienes. Medications not permitted included: long-acting anticholinergics (not allowed within 2 weeks of Visit 1), oral β-adrenergics, β-blockers, cromolyn sodium/nedocromil sodium, and methylxanthines.

### Procedures

Pre-bronchodilator spirometry, body plethysmography, and single-breath diffusing capacity of the lung for carbon monoxide (DL_CO_) were measured according to recommended standards [24]–[26]. Post-bronchodilator spirometry was measured after inhalation of albuterol (400 µg). Predicted normal values for FEV_1_ were calculated using European Community for Steel and Coal equations [27].

Symptom-limited exercise tests were performed on an electronically controlled treadmill using cardiopulmonary exercise testing systems capable of measuring IC. The incremental test in patients with COPD consisted of a 10 W/min protocol, which was repeated at 15 W/min if peak work rate was ≥150 W. Control subjects performed a 15 W/min protocol or repeated the test at 20 W/min if peak work rate was ≥200 W. All tests included a 3-min warm-up (0.27 m/s, 0% grade) followed by stepwise increases in work rate: speed increased linearly from 0.27 to 1.79 m/s, and inclination was calculated based on speed, individual’s weight, and desired work rate [28]. Exercise measurements included: metabolic parameters; minute ventilation 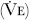; breathing pattern; operating lung volumes; intensity of dyspnea and leg discomfort rated using a modified 10-point Borg scale [29]; and locus of end-exercise symptom limitation (dyspnea, leg discomfort, or both). Maximum breathing capacity (MBC) was estimated as FEV_1_ multiplied by 35 [30], and breathing reserve at end-exercise was calculated as MBC minus peak 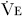.

### Statistical Analysis

The sample size of 100 was dictated by the requirements of the study’s treatment phase. Assuming a 20% dropout rate, 124 patients needed to be randomized to ensure 100 completed the treatment phase, providing 90% power to detect a 100 mL difference in dynamic hyperinflation (change in IC) during exercise from baseline, based on a correlation of 0.6 and a type one error rate of 0.05 using a two-sided test of significance. In the characterization phase, a control group of equal size (n = 100) was included in the study design. Data are presented as means ± standard deviation (SD), unless indicated otherwise. P-values are based on either a two-sample t-test with unequal variance or from a linear regression model, adjusted for age, sex, alcohol status, employment status, and body mass index (smoking status and race were not included in the model because of the small number of participants in some categories). Fisher’s exact test was used to analyze locus of symptom limitation during incremental treadmill exercise. Statistical analyses were applied using SAS version 9.2 software (SAS Institute, Cary, NC, USA).

## Results

### Participants

**Figure 1:**
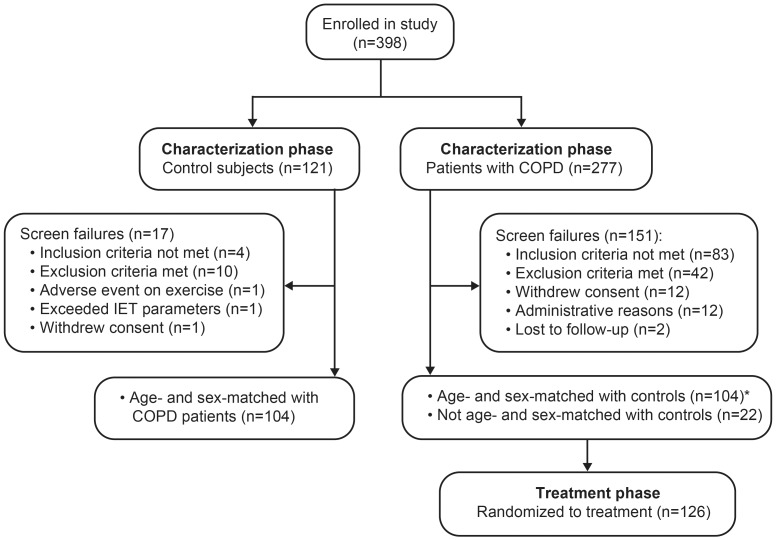
Subject disposition during the characterization phase of the study. IET, incremental exercise testing. *Since 104 control subjects had full characterization data available, only the first 104 patients with COPD, who were eligible for randomization, were age- and sex-matched with the controls for characterization data analysis.

Participants were enrolled into the study between March 16, 2010 and August 1, 2011, the first patient entered the treatment phase on April 14, 2010 and the last completed on November 29, 2011. Participant disposition during the characterization phase is presented in Figure 1. Of the 277 patients with COPD enrolled in the study, 195 met GOLD 1 or 2 spirometric criteria and performed an incremental treadmill exercise test. The reasons for study participant exclusion are given in Figure 1. In total, there were 151 screen failures and 126 patients with COPD eligible for randomization into the treatment phase. In the overall patient population, only 23% (38 out of 164 patients; 17 out of 65 [26%] for GOLD 1 COPD and 21 out of 99 [21%] for GOLD 2 COPD) were excluded for the sole reason of not meeting the operational definition of dynamic lung hyperinflation (i.e., ≥100 mL decrease in IC during at least two out of three of the baseline exercise tests). Of the 121 control subjects enrolled in the study, 104 completed the characterization phase. Only the first 104 patients with COPD, who met the eligibility criteria and were randomized to treatment, were therefore age- and sex-matched with the 104 control subjects for the characterization phase data analysis. Demographic data for the study participants are given in Table 1. The GOLD groups were well matched for baseline demographics. Respiratory medication use at baseline was greater in patients with GOLD 2 compared with GOLD 1 COPD. BDI focal scores were similarly reduced in both GOLD groups compared with controls (p<0.001). The frequency of troublesome respiratory symptoms was similar in the two GOLD groups.

**Table 1:** Subject characteristics.

	GOLD 1	GOLD 2	Control
	n = 41	n = 63	n = 104
Male	21 (51)	27 (43)	48 (46)
Female	20 (49)	36 (57)	56 (54)
Age (years)	59.9±7.7	58.6±8.2	58.8±8.4
Height (cm)	169.7±10.6	167.6±8.3	167.9±11.2
Weight (kg)	78.6±14.6	80.2±13.8	77.6±16.1
Body mass index (kg/m^2^)	27.0±3.8	28.4±3.9	27.4±4.1
Race			
Asian	0 (0)	0 (0)	2 (2)
Black	0 (0)	11 (17)	5 (5)
White	41 (100)	52 (83)	97 (93)
Alcohol status			
Non-drinker	11 (27)	30 (48)	32 (31)
Average consumption†	30 (73)	33 (52)	72 (69)
Employment status			
No	28 (68)	48 (76)	49 (47)
Yes	13 (32)	15 (24)	55 (53)
Time since COPD diagnosis (years)	3.5±3.6	5.1±5.7	N/A
Smoking history (pack-years‡)	46±19***	45±22***	0.9±1.4
Smoking status			
Never smoked	0 (0)	0 (0)	97 (93)
Ex-smoker	23 (56)	26 (41)	7 (7)
Current smokers	18 (44)	37 (59)	0 (0)
Baseline Dyspnea Index focal score	6.9±1.5***	7.1±1.9***	11.4±1.0
Cough/sputum for 3 months for 2 years	27 (66)	42 (67)	N/A
Most troublesome respiratory symptom			
Shortness of breath	29 (71)	44 (70)	N/A
Cough	8 (20)	9 (14)	N/A
Wheezing	0 (0)	5 (8)	N/A
Sputum	4 (10)	3 (5)	N/A
Other	0 (0)	2 (3)	N/A
Respiratory medications at baseline			
Long-acting β_2_-agonist	1 (2)	9 (14)	0 (0)
Inhaled corticosteroid	0 (0)	8 (13)	0 (0)
Long-acting β_2_-agonist/inhaled corticosteroid combination	0 (0)	6 (10)	0 (0)
Anticholinergics	3 (7)	8 (13)	0 (0)
Short-acting muscarinic antagonist	2 (5)	4 (6)	0 (0)
Long-acting muscarinic antagonist(tiotropium)	1 (2)	5 (8)	0 (0)

Data are presented as mean ± standard deviation for continuous variables and number of patients (%) for categorical variables.

GOLD, Global Initiative for Chronic Obstructive Lung Disease; N/A, not applicable.

***p<0.001 versus control, using a two-sample *t*-test with unequal variance.

† Drinks alcohol, but to an extent that would not interfere with participation in the trial.

‡ 1 pack-year represents 20 cigarettes per day for 1 year.

### Pulmonary Function

Pulmonary function data are shown in Table 2. Both GOLD groups had a significantly greater functional residual capacity (FRC) and residual volume (RV) compared with controls (p<0.001). Compared with controls, IC and slow vital capacity (SVC) at rest were significantly lower in patients with GOLD 2 COPD (p<0.001), but not in patients with GOLD 1 COPD. Specific airway resistance was significantly higher (p<0.001) and DL_CO_ significantly lower (p<0.001) in both GOLD groups compared with controls.

**Table 2:** Pulmonary function.

	GOLD 1	GOLD 2	Control
	n = 41	n = 63	n = 104
FEV_1_ post-bronchodilator (L)	2.65±0.75***	1.84±0.46*** ^,^ ###	3.11±0.81
FEV_1_ post-bronchodilator (% predicted)	92±10***	67±9*** ^,^ ###	112±15
FEV_1_/FVC post-bronchodilator (%)	64±5***	56±8*** ^,^ ###	81±5
Pre-bronchodilator (Visit 1)			
FEV_1_ (L)	2.45±0.71***	1.67±0.48*** ^,^ ###	3.02±0.80
FEV_1_ (% predicted)	86±12***	60±11*** ^,^ ###	109±15
FEV_1_/FVC (%)	61±6***	55±9*** ^,^ ###	78±6
SVC (L)	4.04±1.20	3.23±0.87*** ^,^ ###	3.99±1.04
IC (L)	2.90±1.01	2.35±0.64*** ^,^ ###	2.86±0.84
IC (% predicted)	103±22	89±16*** ^,^ ###	106±18
FRC (L)	3.77±0.91***	3.65±0.81***	2.95±0.76
FRC (% predicted)	117±24***	118±26***	93±18
RV (L)	2.63±0.81***	2.77±0.72*** ^,^ #	1.81±0.58
RV (% predicted)	121±38***	130±35*** ^,^ #	84±24
TLC (L)	6.67±1.63***	6.01±1.11** ^,^ #	5.77±1.36
TLC (% predicted)	111±16***	105±14***	99±12
sR_aw_ (cmH_2_O/L/s)†	12.0±5.7***	14.8±10.9*** ^,^ #	7.2±4.7
sR_aw_ (% predicted)†	292±144***	363±267*** ^,^ #	176±120
DL_CO_ (mL/min/mmHg)‡	18.6±6.7***	16.0±4.6*** ^,^ #	22.9±6.7
DL_CO_ (% predicted)‡	73±20***	65±16*** ^,^ #	92±17

Data are presented as mean ± standard deviation.

GOLD, Global Initiative for Chronic Obstructive Lung Disease; FEV_1_, forced expiratory volume in 1 s; FVC, forced vital capacity; SVC, slow vital capacity; IC, inspiratory capacity; FRC, functional residual capacity; RV, residual volume; TLC, total lung capacity; sR_aw_, specific airway resistance; DL_CO_, diffusing capacity of the lung for carbon monoxide.

P-values are from a linear regression model, adjusted for age, sex, alcohol status, employment status, and body mass index.

**p<0.01 versus control;

***p<0.001 versus control.

# p<0.05 GOLD 1 versus GOLD 2;

### p<0.001 GOLD 1 versus GOLD 2.

† GOLD 1, n = 40; GOLD 2, n = 62.

‡ GOLD 2, n = 61.

### Incremental Exercise

**Figure 2:**
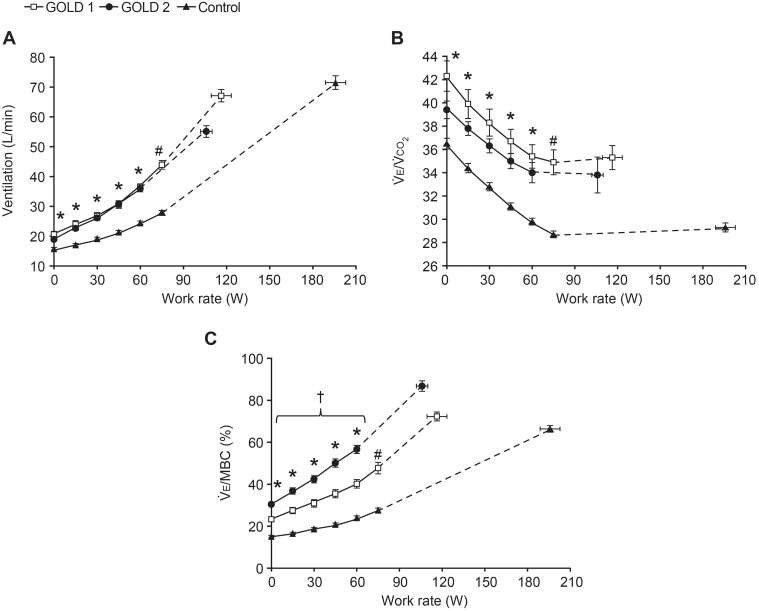
Ventilatory parameters versus work rate during incremental treadmill exercise. (A) Minute ventilation 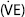, (B) ventilatory equivalent for carbon dioxide 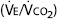, and (C) ventilation as a fraction of estimated maximum breathing capacity 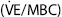. Data are shown as mean ± standard error. Data plotted at 0 watts are warm-up data (0.27 m/s), followed by points at standardized work rates and at peak exercise. *p<0.05 GOLD 1 and 2 versus controls at a standardized work rate; ^#^p<0.05 GOLD 1 versus controls; ^†^p<0.05 GOLD 2 versus controls (confidence intervals do not overlap indicating p<0.05).

Peak exercise data are shown in Table 3. Peak work rate was significantly lower in both GOLD groups compared with controls (p<0.001), but was similar in GOLD 1 and 2. Peak oxygen uptake 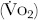 and carbon dioxide production 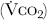 were significantly lower in both GOLD groups compared with controls (p<0.001), and were significantly lower in GOLD 2 compared with GOLD 1 (p<0.05). Peak 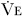 was significantly reduced in GOLD 2 compared with the GOLD 1 and control groups (p<0.001), but GOLD 1 was similar to the controls. Both GOLD groups had significantly greater 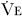 and ventilatory equivalents for carbon dioxide 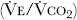 at rest and at any given work rate during exercise compared with controls (Figure 2). 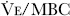 at rest and throughout exercise was also significantly greater in both GOLD groups compared with controls, and was higher in GOLD 2 compared with GOLD 1 (Figure 2). Minimum oxygen saturations were not significantly different in GOLD 1 compared with GOLD 2 and controls, but were lower by 1.4% in GOLD 2 compared with controls (p<0.01) (Table 3).

**Table 3:** Peak symptom-limited incremental treadmill exercise.

	GOLD 1	GOLD 2	Control
	n = 41	n = 63	n = 104
Work rate (W)	116±42***	106±31***	196±67
Dyspnea Borg scale†	5.6±2.5	5.5±2.4	5.0±2.8
Leg discomfort Borg scale†	4.7±2.8	5.3±2.6	4.7±2.8
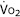 (L/min)†	1.81±0.59***	1.62±0.41*** ^,^ #	2.17±0.70
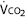 (L/min)†	1.94±0.71***	1.69±0.47*** ^,^ #	2.46±0.79
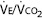 †	35.3±6.6***	33.8±12.1**	29.3±3.9
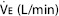 †	67.1±23.6	55.1±15.4*** ^,^ ###	71.5±23.4
Breathing reserve (L/min)†	25.6±15.4***	9.6±14.3*** ^,^ ###	37.3±19.9
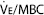 (%)†	72±14	87±20*** ^,^ ###	66±16
Vt (L)†	1.92±0.59***	1.57±0.44*** ^,^ ###	2.17±0.66
*F*b (breaths/min)†	35.4±7.6	35.7±7.3	33.4±6.6
Ti/Ttot †	0.44±0.05	0.41±0.06*** ^,^ ##	0.45±0.06
IC (L)	2.67±0.78**	2.17±0.59*** ^,^ ###	2.90±0.81
ΔIC peak–rest (L)	–0.52±0.44***	–0.42±0.31***	0.01±0.39
IRV (L)†	0.75±0.40	0.60±0.40*	0.73±0.36
EELV (L)	4.00±1.11***	3.85±0.87***	2.87±0.78
SpO_2 _minimum (%)†	94.4±2.8	93.5±2.9**	94.9±2.7

Data are presented as mean ± standard deviation.

GOLD, Global Initiative for Chronic Obstructive Lung Disease; 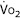, oxygen uptake; 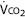, carbon dioxide production; 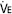, minute ventilation; MBC, maximum breathing capacity; Vt, tidal volume; *F*b, breathing frequency; Ti, inspiratory time; Ttot, total respiratory time; Ti_/_Ttot, inspiratory duty cycle; IC, inspiratory capacity; Δ, change; IRV, inspiratory reserve volume; EELV, end-expiratory lung volumes; SpO_2_, arterial oxygen saturation measured by pulse oximetry.

P-values are from a linear regression model, adjusted for age, sex, alcohol status, employment status, and body mass index.

*p<0.05 versus control;

**p<0.01 versus control;

***p<0.001 versus control

# p<0.05 GOLD 1 versus GOLD 2;

## p<0.01 GOLD 1 versus GOLD 2;

### p<0.001 GOLD 1 versus GOLD 2.

† GOLD 2, n = 62.

**Figure 3:**
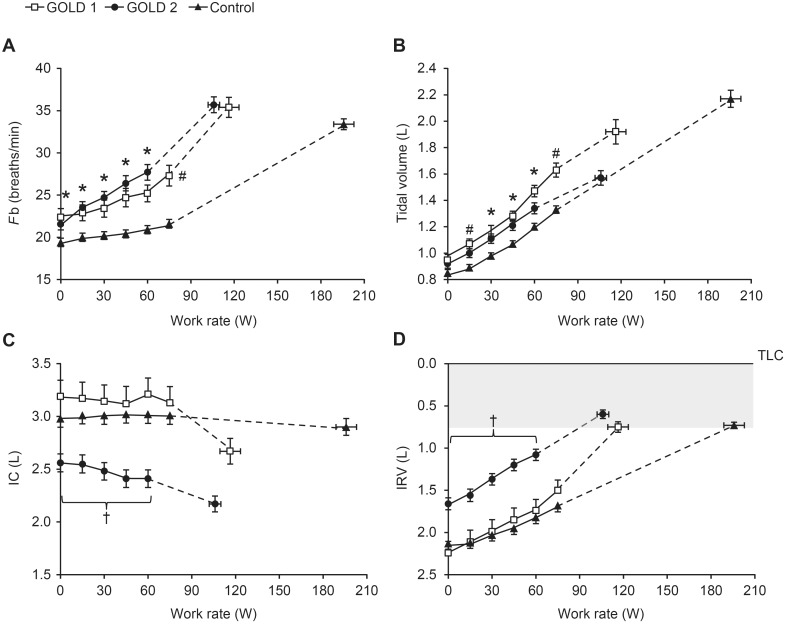
Breathing pattern and operating lung volume measurements versus work rate during incremental treadmill exercise. (A) Breathing frequency (*F*b), (B) tidal volume, (C) inspiratory capacity (IC), and (D) inspiratory reserve volume (IRV) showing that all three groups reach a similar minimal value at end-exercise (shaded area). Data are shown as mean ± standard error. TLC, total lung capacity. *p<0.05 GOLD 1 and 2 versus controls at a standardized work rate; ^#^p<0.05 GOLD 1 versus controls; ^†^p<0.05 GOLD 2 versus GOLD 1 and controls (confidence intervals do not overlap indicating p<0.05).

**Figure 4:**
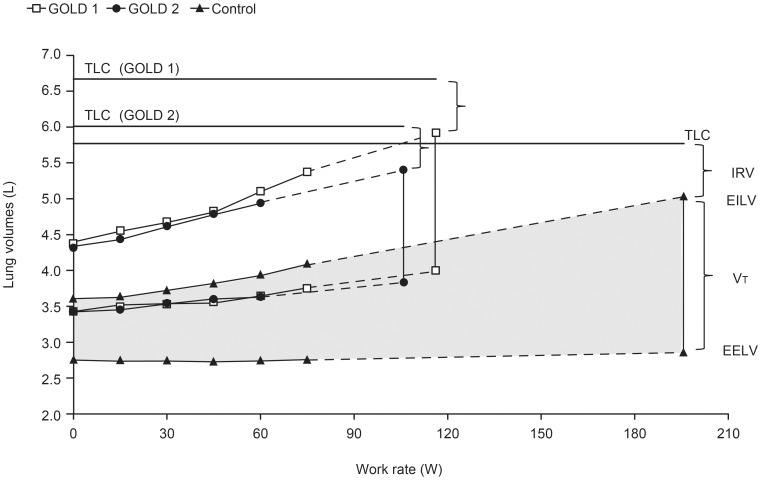
Operating lung volumes versus work rate during incremental treadmill exercise. End-expiratory lung volume (EELV) and end-inspiratory lung volume (EILV) measurements were significantly greater (p<0.05) throughout exercise in GOLD 1 and 2 compared with controls (p-values based on two-sample t-test with unequal variance). Data are shown as means. TLC, total lung capacity; IRV, inspiratory reserve volume; VT, tidal volume.

In accordance with subject inclusion criteria, dynamic hyperinflation was shown in both GOLD groups as a significant decrease in IC from rest to peak exercise; whereas, the controls did not change IC significantly during exercise (Table 3). IC values at rest and throughout exercise were similar in the GOLD 1 and control groups, but were significantly smaller in the GOLD 2 group (Figure 3). The GOLD 2 group also had a significantly smaller inspiratory reserve volume (IRV) than both the GOLD 1 and control groups at rest and at a given work rate during exercise, but all groups reached a similar IRV at end-exercise (Figure 3). End-expiratory lung volumes (EELV) and end-inspiratory lung volumes (EILV) were significantly greater in patients with COPD at rest and throughout exercise compared with controls (Figure 4).

**Figure 5:**
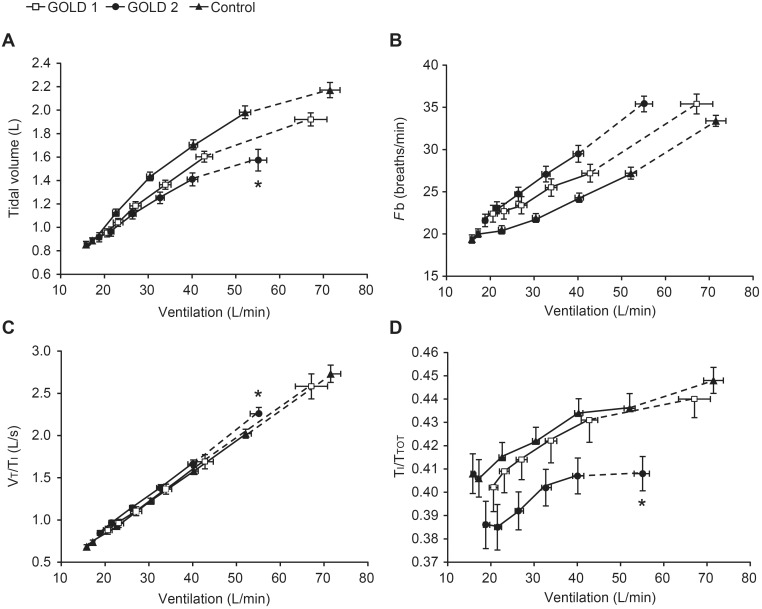
Breathing pattern measurements versus minute ventilation during incremental treadmill exercise. (A) Tidal volume, (B) breathing frequency (*F*b), (C) mean inspiratory flow (tidal volume/inspiratory time [Vt/Ti]), and (D) inspiratory duty cycle (inspiratory time/total respiratory time [Ti/Ttot]). Data are shown as mean ± standard error. *p<0.05 GOLD 2 versus GOLD 1 and controls at peak exercise (confidence intervals do not overlap indicating p<0.05).

To evaluate breathing pattern differences across groups, measurements were plotted against 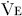 (Figure 5). There was a significantly smaller tidal volume (Vt) response in GOLD 2 compared with the GOLD 1 and control groups at peak exercise, which was attributable to the smaller IC in the GOLD 2 group (Table 3). The inspiratory duty cycle (inspiratory time/total respiratory time [Ti/Ttot]) at rest and throughout exercise was also lower in GOLD 2 compared with the GOLD 1 and control groups.

**Figure 6:**
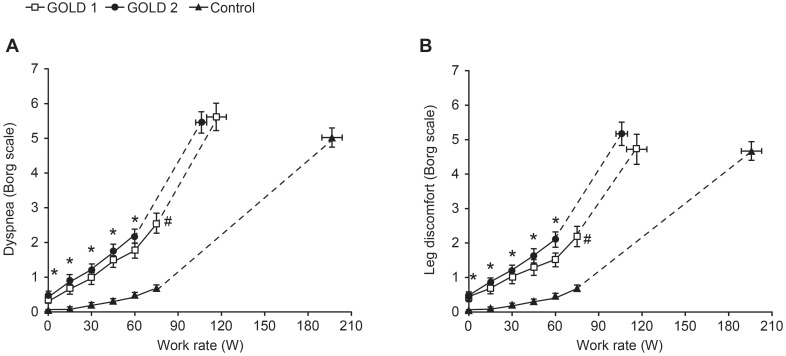
Intensity ratings of (A) dyspnea and (B) leg discomfort versus work rate during incremental treadmill exercise. Data are shown as mean ± standard error. *p<0.05 GOLD 1 and GOLD 2 versus controls at a standardized work rate; ^#^p<0.05 GOLD 1 versus controls (confidence intervals do not overlap indicating p<0.05).

There were no significant differences across the groups in peak symptom intensity ratings for dyspnea and leg discomfort (Table 3); however, both GOLD groups had consistently higher values for these parameters at a given work rate compared with controls (Figure 6). There was a significant difference in the distribution of reasons for stopping exercise across the groups (p<0.001). There was a higher frequency of dyspnea, either alone or in combination with leg discomfort in the GOLD 1 (88% [36/41]) and GOLD 2 (81% [51/63]) groups compared with controls (63% [65/104]) and a higher frequency of no dyspnea or leg discomfort in the controls (14% [15/104] compared with the GOLD 1 (2% [1/41]) and GOLD 2 (0% [0/63]) groups.

## Discussion

The main findings of this study are: (1) compared with healthy participants, symptomatic patients with mild-to-moderate COPD and measurable dynamic hyperinflation showed distinct physiological impairment at rest and during exercise; (2) the magnitude of dyspnea, leg discomfort, and exercise limitation was similar in GOLD 1 and 2 COPD; (3) higher ventilatory requirements and greater dynamic respiratory mechanical abnormalities were present in both GOLD groups compared with controls; and 4) increased constraints on Vt expansion during exercise were present in GOLD 2 COPD, in association with a reduced resting IC in this group.

Healthy participants and patients with COPD were well matched for age, sex, and BMI. Subjects in both GOLD groups had a history of heavy smoking and reported moderate-to-severe chronic dyspnea. Only a minority of patients received regular pharmacotherapy for COPD. Of the three major respiratory symptoms, the majority of patients reported that dyspnea was the most troublesome (Table 1).

Contrary to our expectation, the nature and extent of lung function impairment were remarkably similar in the two GOLD groups. Both groups showed increased pulmonary gas trapping (RV), increased lung hyperinflation (FRC) and reduced DL_CO_, reflecting increased airway closure, alterations in the elastic properties of the respiratory system and/or delayed mechanical time constants for lung emptying, and disruption of the alveolar–capillary surface area for gas exchange, respectively. However, in contrast to patients with GOLD 2 COPD, those with GOLD 1 COPD had preserved resting IC and SVC. Thus, total lung capacity (TLC), FRC, and RV increased in parallel in GOLD 1 COPD, whereas FRC and RV increased more relative to TLC in GOLD 2 COPD. This preservation of IC and SVC in those with milder airway obstruction (as measured by FEV_1_) has previously been reported in cross-sectional studies in COPD [10], [11], [31], [32], and may have important implications for ventilatory capacity during exercise and response to therapy.

Peak work rate and 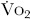 during incremental treadmill exercise were reduced in both the GOLD groups compared with controls. This magnitude of reduction in exercise performance in the GOLD groups is considered clinically meaningful [33]. Ventilatory requirements were increased to a similar extent in the GOLD groups compared with controls, by 5–10 L/min at a standardized submaximal work rate, but represented a greater proportion of MBC in patients with GOLD 2 COPD. The cause of the additional ventilatory stimulation in COPD was not determined. Many patients in both GOLD groups had physiological features of emphysema (i.e., reduced DL_CO_ and increased FRC), which is known to be associated with disruption of pulmonary vascular beds. 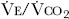 was elevated at rest and throughout exercise in the GOLD groups compared with controls, suggesting increased inefficiency of CO_2_ elimination related to an increased physiological dead space. However, arterial partial pressure of CO_2_ measurements would be required to evaluate such abnormalities of pulmonary gas exchange more accurately. In accordance with the study of Barbera et al. [13], there was no significant arterial O_2_ desaturation at peak exercise in either GOLD group. Other possible contributors to increased 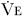, such as earlier metabolic acidosis due to skeletal muscle deconditioning (with increased afferent sensory inputs), reduced inspiratory muscle strength, or occult cardiovascular impairment, were not quantified in this study [34]–[37].

In accordance with the selection criteria, all study patients with COPD showed evidence of dynamic lung hyperinflation during exercise. This criterion was met in 77% of those originally screened, who met all the other inclusion criteria, i.e., 23% of otherwise eligible patients were excluded because of a lack of dynamic hyperinflation. Breathing pattern alterations during exercise were more pronounced in the GOLD 2 group because of the decreased resting IC. This group had a relatively diminished breathing reserve as crudely measured by the MBC minus peak 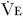 difference. Moreover, such patients reached a minimal IRV of 0.6 L and a Vt/IC ratio exceeding 70% at a peak 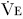 of only 55 L/min. The reduced Vt expansion in the GOLD 2 group compared with the GOLD 1 group and controls was associated with a reduced Ti/Ttot and lack of a compensatory increase in mean inspiratory flow rate. It is not clear if these differences in breathing pattern reflect the imposed restrictive mechanics because of high lung volumes, central neural inhibition of the drive to breathe as a result of the vagal inflation reflex, a compensation strategy to minimize discomfort associated with increased elastic loading, shallow breathing as a result of increased inspiratory muscle dysfunction [37], or some combination of these. The low IRV at a relatively low peak 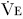 and power output in patients with GOLD 2 COPD, indicates that EILV was positioned close to TLC and the upper alinear extreme of the respiratory system’s pressure–volume relation where elastic loading of respiratory muscles is increased. Collectively, our results suggest that critical mechanical constraints and the associated severe breathing discomfort at a relatively low peak 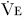 may have contributed to exercise limitation in patients with GOLD 2 COPD. The preservation of resting IC in patients with GOLD 1 COPD compared with those with GOLD 2 COPD, allowed greater Vt expansion and the attainment of a significantly higher peak 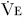 before the onset of intolerable dyspnea.

At the limits of tolerance, all groups reported severe dyspnea and leg discomfort, although these symptoms occurred at lower peak work rates in the GOLD groups compared with controls. Dyspnea and leg discomfort ratings were increased throughout exercise in both GOLD groups compared with controls. Previously, we postulated that increased dyspnea in patients with mild-to-moderate COPD compared with healthy individuals ultimately reflects the increased contractile respiratory muscle effort (and increased central corollary discharge) associated with increased 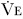 and the increased resistive and elastic loads on the respiratory muscles [11], [17]. It is noteworthy that leg discomfort intensity ratings were broadly similar in magnitude to the dyspnea intensity ratings in both GOLD groups and were higher than in the control group for a given work rate. This was unexpected as perceived leg discomfort is thought to be less problematic during treadmill exercise compared with cycle exercise [38]. One potential reason for the higher than expected ratings of leg discomfort in this study could be the relatively steep inclination that subjects were required to walk using this incremental exercise protocol, especially those with lower body weights. Alternatively (or in addition), there is increasing evidence of measurable peripheral muscle dysfunction in patients with COPD who have mild-to-moderate airway obstruction [39], [40].

Our strict selection criteria (i.e., the requirement for dynamic hyperinflation to occur) could mean that our results may not be generalizable to the broader COPD population with milder airway obstruction, many of whom may be less symptomatic than our study subjects. However, study exclusion solely due to a lack of significant dynamic hyperinflation during exercise occurred in only 23% of those meeting GOLD 1 or 2 spirometric criteria that performed the baseline exercise tests. A focus on symptomatic patients who have documented impairment of respiratory mechanics is justified, as this was the primary outcome measure of interest in the subsequent intervention phase of the study. The lack of a detailed evaluation of pulmonary gas exchange in this study means that the mechanism of the increased ventilatory demand in COPD remains unresolved.

In conclusion, this study is the first to elucidate the nature and extent of physiological impairment during treadmill exercise in a sizeable population of symptomatic patients with mild-to-moderate COPD who have evidence of air trapping. Thus, it provides new insights into mechanisms of poor exercise tolerance in this group. Consistent ventilatory abnormalities during exercise in both GOLD groups included higher ventilatory requirements for a given power output and greater dynamic mechanical constraints. Lower resting IC in the GOLD 2 group was associated with altered breathing pattern responses during exercise with attainment of minimal dynamic IRV (and associated severe dyspnea) at a significantly lower peak 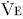 than the GOLD 1 and control groups. Thus, in the GOLD 2 group, the respiratory system had approached or reached its physiological limits at the termination of exercise. It remains to be determined whether bronchodilator treatment, which improves respiratory mechanics, can increase exercise tolerance in this little-studied population.

## Supporting Information

Checklist S1 CONSORT checklist.
(DOC)

Protocol S1 Trial protocol.
(PDF)
